# Clinical validity of the Pediatric Assessment Triangle in a pediatric emergency department

**DOI:** 10.3389/fped.2025.1435604

**Published:** 2025-05-13

**Authors:** Siqi Zhu, Yufen Wu, Biyan Yu, Biyu Shen, Linjie Fang, Biru Li, Long Xiang

**Affiliations:** ^1^ Department of Pediatric Critical Care Medicine, Shanghai Children's Medical Center, School of Medicine, Shanghai Jiao Tong University, Shanghai, China; ^2^ Department of Nursing, Shanghai Children's Medical Center, School of Medicine, Shanghai Jiao Tong University, Shanghai, China; ^3^ Department of Pediatric Emergency Medicine, Shanghai Children's Medical Center, School of Medicine, Shanghai Jiao Tong University, Shanghai, China

**Keywords:** Pediatric Assessment Triangle, pediatric emergency department, emergency triage system, pediatric emergency overcrowding, pediatric

## Abstract

**Objectives:**

This study aimed to evaluate the clinical validity of the Pediatric Assessment Triangle (PAT) in a pediatric emergency department (PED).

**Methods:**

We conducted a retrospective analysis of 799 children who visited our PED and collected data on age, sex, disease severity, expense, and disposition. We analyzed the correlations between PAT and disease, age, waiting time, disposition, and cost.

**Results:**

In total, 429 boys (53.7%) and 370 girls (46.3%), with an average age of 4.97 years, were enrolled. The number of children in levels 2, 3, and 4 was 5 (0.6%), 158 (19.8%), and 636 (79.6%), respectively. Respiratory system diseases comprised 78.7% of all disease cases. The top three highest proportions of critical cases were endocrine system diseases (100%), toxic exposure (50%), and circulatory system diseases (40%). Children aged 3–8 years accounted for 45.3% of the cases. The incidence of critical cases was most prevalent within the neonatal population (21.4%), followed by children aged 8–15 years (2.1%) and 3–8 years (0.6%). The area under the receiver operating characteristic curve for the PAT in predicting hospitalization was 0.966. The mean waiting time for level-2 children was 3.80 min.

**Conclusions:**

As a tool used for PED triage, the PAT can specifically identify critical cases, particularly in recognition of pediatric respiratory emergencies and neonatal emergencies, and demonstrates significant superiority. Future multicenter studies should be conducted in pediatric emergency medical centers to investigate the effectiveness of PAT in PED triage further.

## 1 Background

The number of pediatric emergency department (PED) visits has risen with societal development and the increase in healthcare demand. The 2018 National Hospital Ambulatory Medical Care Survey (USA) reported 130 million emergency department visits annually, with 25.6 million visits made by children under the age of 15 (1). The overcrowding in PEDs has thereby exceeded the rate of increase in pediatric healthcare professionals (2). This overcrowding leads to excessively long waiting times and inadequate monitoring conditions in waiting areas, increasing safety risks for patients. These scenarios may result in worsened clinical conditions, delayed treatment and diagnosis, and even patients leaving the hospital before being seen by a doctor (3). Additionally, patients in the PED visit in high volume and during concentrated visiting hours, with an acute onset and rapid disease progression. Moreover, these children often show poor self-expression capabilities, manifest irritability, and may be surrounded by anxious family members. However, only approximately 20% of children with acute and severe diseases are brought to the PED for emergency medical treatment (4). Therefore, there is a need for a rapid assessment tool to assist triage nurses in accurately distinguishing critically ill children from those in urgent need of emergency medical treatment (5).

Emergency triage refers to the rapid classification of emergency patients based on the severity of their illness, the principle of treatment priority, and the rational utilization of emergency medical resources to determine priority treatment for patients in need of urgent care (6). Commonly used triage scales in PEDs currently include the Australasian Triage Scale (ATS), Emergency Severity Index (ESI), Manchester Triage System (MTS), Canadian Triage and Acuity Scale (paedCTAS), and Pediatric Assessment Triage (PAT). Although researchers have reported the effectiveness and reliability of these triage scales in pediatric emergency services (5, 7), there is currently no internationally standardized tool for pre-triage assessment of pediatric emergency patients. The principal reason for this reflects the disparities in clinical parameters among different age groups and that critically ill children may initially appear stable but then rapidly deteriorate. Therefore, in the initial stages, triage tools may not provide sufficient warning to ensure adequate medical intervention (8, 9).

The PAT is a rapid assessment tool introduced by the American Academy of Pediatrics in 2000. This tool allows emergency healthcare providers to assess the overall presentation of sick children without the need for equipment. Within 30–60 s, visual and auditory assessments of appearance, breathing, and circulation are conducted to evaluate the child's oxygenation, ventilation, perfusion, and neurological function; determine the severity of their clinical presentation; and identify the type of urgent intervention required (10). In this study, we combined the characteristics of pediatric emergency patients, the number of medical staff, and the allocation of medical resources at the Shanghai Children's Medical Center (SCMC) to propose a five-level triage based on the PAT. We conducted a retrospective analysis of 799 cases of pediatric emergency visits to investigate the effectiveness of the PAT in the early identification of critically ill children in the pre-triage stage at our center.

## 2 Materials and methods

### 2.1 Data source

The present investigation was a single-center retrospective analysis in which we randomly selected 799 pediatric patients who visited the PED of the SCMC in September 2023. The age range of the patients included in the study was from birth to 18 years, and the PED triage nurse conducted a PAT evaluation for each patient upon their arrival. The PAT, introduced from Boston Children's Hospital, has been applied and promoted in the PED in our medical center for 10 years. The PED nurse met the following requirements to work in triage: at least 6 months of uninterrupted PED work experience, and training in basic pediatric assessment, triage concepts, and practices. The triage area was equipped with a separate room staffed by a trained nurse and a nursing assistant. This study was approved by the Ethics Committee of Shanghai Children's Medical Center (Shanghai, China, approval number: SCMCIRB-K2023222-1). As this was a retrospective study and the children did not receive any intervention, written informed consent was not applicable.

### 2.2 Study methods

#### 2.2.1 Pediatric Assessment Triangle assessment

**Figure 1:**
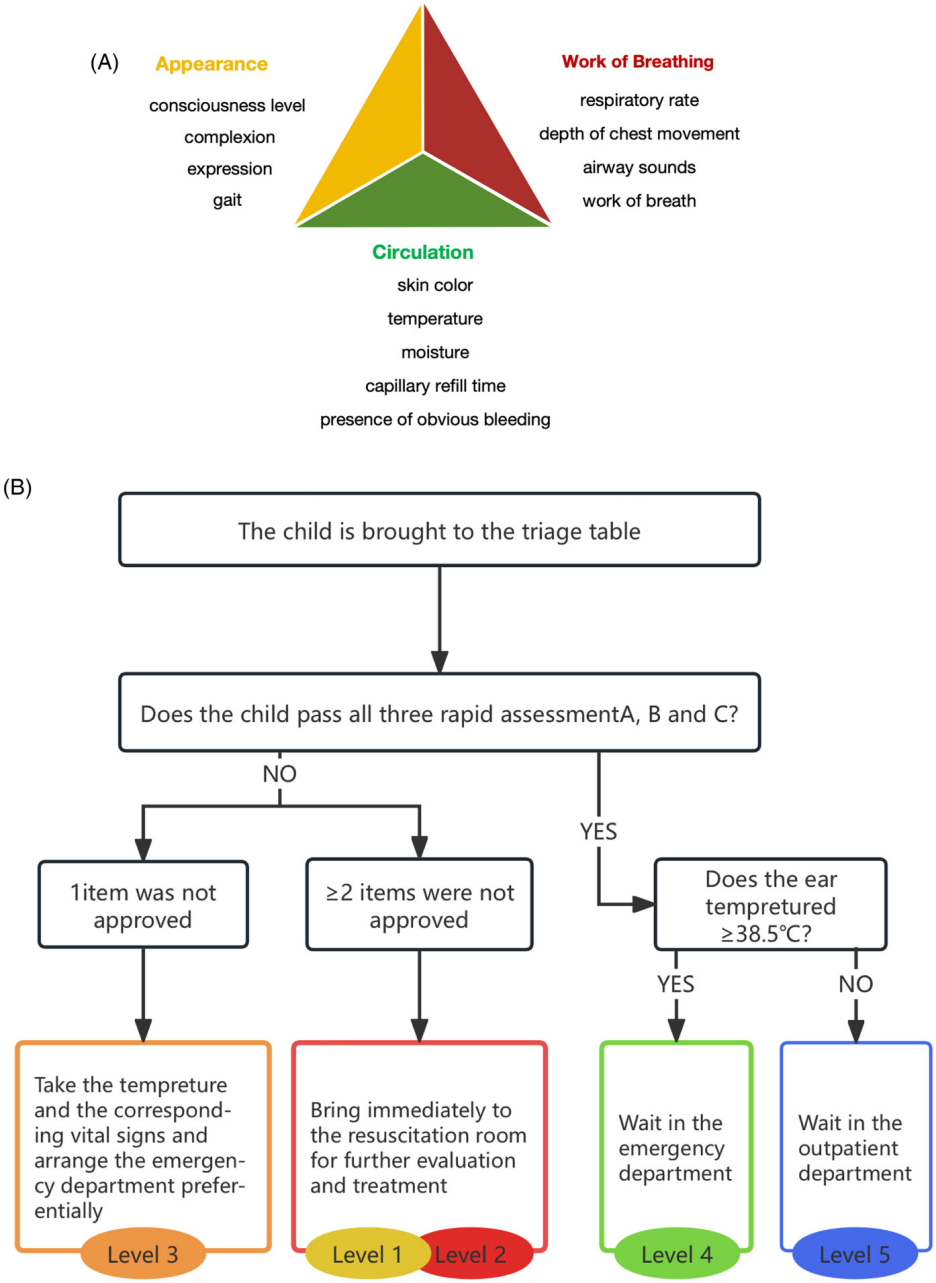
**(A)** Pediatric Assessment Triangle. **(B)** Five-level triage system based on the Pediatric Assessment Triangle.

The formally trained triage nurse conducted the PAT assessment, which covered three aspects: appearance, work of breathing, and circulation. The appearance aspect included the patient's level of consciousness, complexion, expression, and gait; the work of breathing aspect included respiratory rate, depth of chest movement, airway sounds, and work of breath; and circulation included skin color, temperature, moisture, capillary refill time, and presence of obvious bleeding (Figure 1A).

#### 2.2.2 Five-level triage based on the Pediatric Assessment Triangle

Based on the three items of appearance, work of breathing, and circulation, pediatric patients in the PED pre-triage were divided into five levels. Levels 1 and 2 were critical cases that required immediate intervention in the resuscitation room, level 3 comprised urgent cases that required priority treatment, level 4 entailed semi-urgent cases with stable vital signs and were treated in order, and level 5 constituted non-urgent patients who were redirected to outpatient treatments. The specific pre-triage scheme was as follows: if the patient failed to pass at least two items in the PAT, they were classified as level 1 (blue alert) or 2 (red alert); if they failed one item, they were classified as level 3 (yellow alert); if they passed all three items and their body temperature was ≥38.5°C, the patient was classified as level 4 (green alert); and if their body temperature was <38.5°C, the patient was classified as level 5 (Figure 1B).

#### 2.2.3 Outcomes

The primary outcome was the proportion of critically ill children with different diseases. We defined critical cases as levels 1 and 2 in the pre-triage, and secondary outcomes included age, destination, waiting time, and medical expenses.

#### 2.2.4 Statistical methods

Descriptive statistics such as mean, maximum, and minimum values were used for counting data, while percentages were used for continuous data. Frequencies and percentages were applied to describe categorical variables. A *P*-value of <0.05 was considered to be statistically significant. We adopted the area under the receiver operating characteristic (ROC) curve (AUROC) and the area under the precision-recall curve (AUPRC) by logistic function to describe the effectiveness of the variables in predicting outcomes. SPSS 25.0 statistical software and Python 3.10 were used for data processing.

## 3 Results

### 3.1 Baseline characteristics

**Figure 2:**
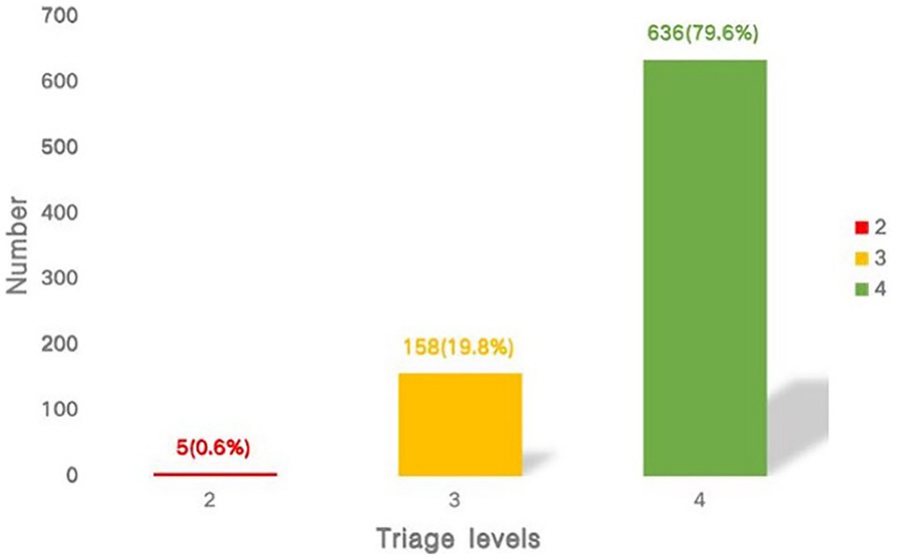
The number of patients in levels 2, 3, and 4.

This analysis encompassed 799 cases, with 429 boys (53.7%) and 370 girls (46.3%). The mean age was (4.97 ± 3.62) years, with approximately 75.7% of the children under 8 years old. The number of patients in levels 2, 3, and 4 was 5 (0.6%), 158 (19.8%), and 636 (79.6%), respectively. The diagnosis and general information of the patients are presented in Table 1, and the results of the PAT pre-triage are shown in Figure 2.

**Table 1:** Baseline characteristics of study population (*n* = 799).

Characteristic	Classification	Number of cases (%)	Number of critical cases (%)
Gender			
	Female	370 (46.3)	6 (1.6)
Age			
	Neonate	14 (1.8)	3 (21.4)
	1–12 months	81 (10.1)	0 (0.0)
	13–36 months	185 (23.2)	0 (0.0)
	3 years < age < 8 years	362 (45.3)	2 (0.6)
	8 years ≤ age < 15 years	145 (18.1)	3 (2.1)
	≥15 years	12 (1.5)	0 (0.0)
Category of illness			
	Respiratory disease	629 (78.7)	1 (0.2)
	Gastrointestinal disease	98 (12.3)	1 (1.0)
	Accidental damage	24 (3.0)	0 (0.0)
	Neurological disease	13 (1.6)	1 (7.7)
	Infectious disease	11 (1.4)	1 (9.1)
	Allergic disease	7 (0.9)	0 (0.0)
	Urological disease	5 (0.6)	0 (0.0)
	Circulatory disease	5 (0.6)	2 (40.0)
	Hematological disease	2 (0.3)	0 (0.0)
	Drug poisoning	2 (0.3)	1 (50.0)
	Others	2 (0.3)	0 (0.0)
	Endocrine disease	1 (0.1)	1 (100.0)
Triage level			
	1	0 (0.0)	0 (0.0)
	2	5 (0.6)	5 (100.0)
	3	158 (19.8)	3 (1.9)
	4	636 (79.6)	0 (0.0)
	5	0 (0.0)	0 (0.0)

### 3.2 The Pediatric Assessment Triangle and the primary outcome

**Figure 3:**
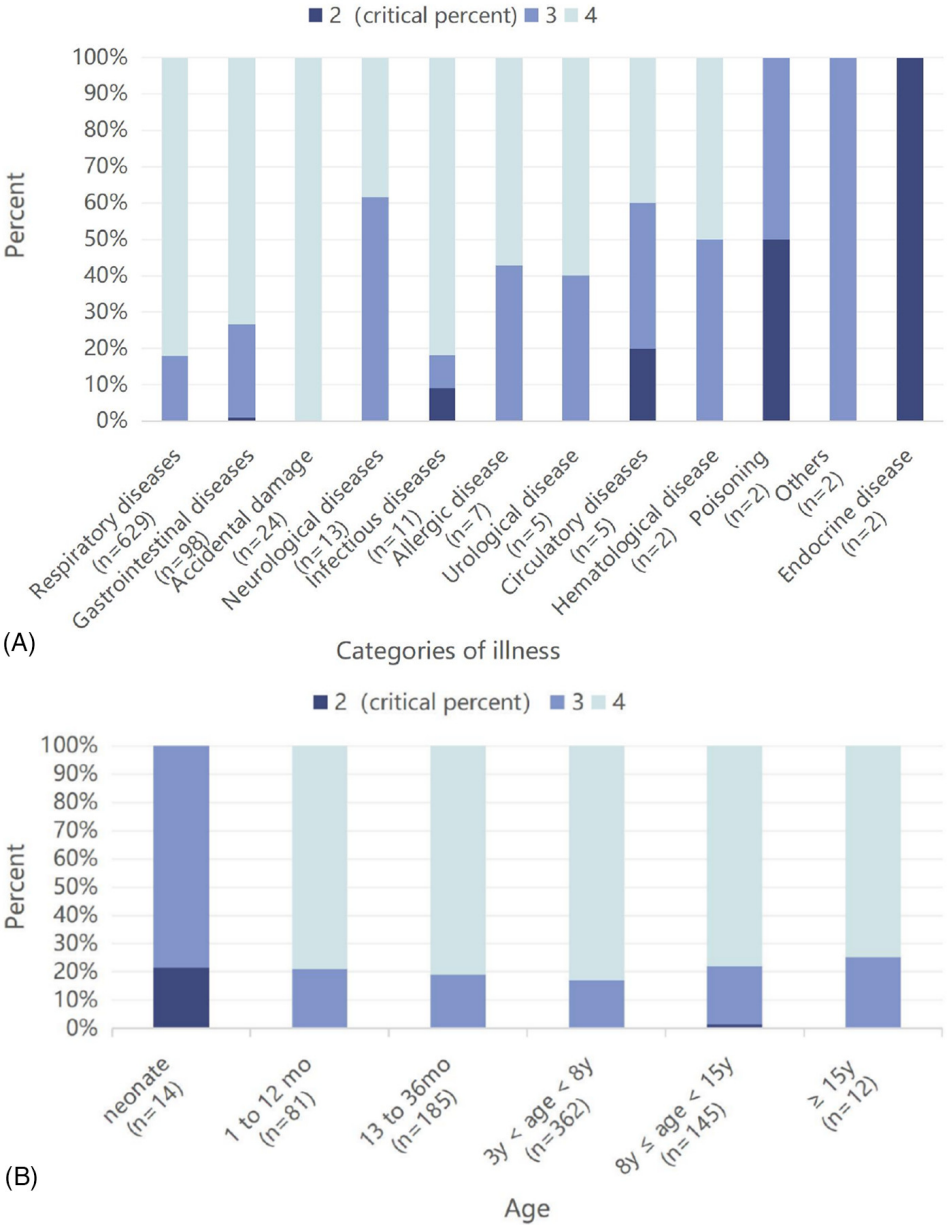
**(A)** The most common types of diseases in our pediatric emergency department and the proportion of critically ill cases among the cases with different types of diseases. **(B)** The number of patients in different age groups and the proportion of critically ill cases in the different age groups.

The correlation between the PAT and the severity of different systemic diseases varied. The number, proportion, and corresponding proportion of critically ill children with different systemic diseases are shown in Table 1. The most common type of disease in our PED was respiratory system diseases (78.7%), while non-respiratory system diseases only accounted for 21.3%. Among the different types of diseases, the top three highest proportions of critically ill cases (non-exclusive) were endocrine system diseases (100%), toxic exposure (50%), and circulatory system diseases (40%) (Figure 3A).

### 3.3 The Pediatric Assessment Triangle and the secondary outcomes

The correlations between PAT and age are shown in Table 1, including the number of pediatric patients and the corresponding number of critically ill patients in each age group. Of these, the 3–8-year-old group had the highest number of patients (45.3%) (Figure 3B). The proportion of critically ill patients was highest among the neonates (21.4%), followed by the 8–15-year-olds (2.1%) and 3–8-year-olds (0.6%) (Figure 3B).

### 3.4 Other outcomes

#### 3.4.1 Correlation between the Pediatric Assessment Triangle and hospitalization

**Figure 4:**
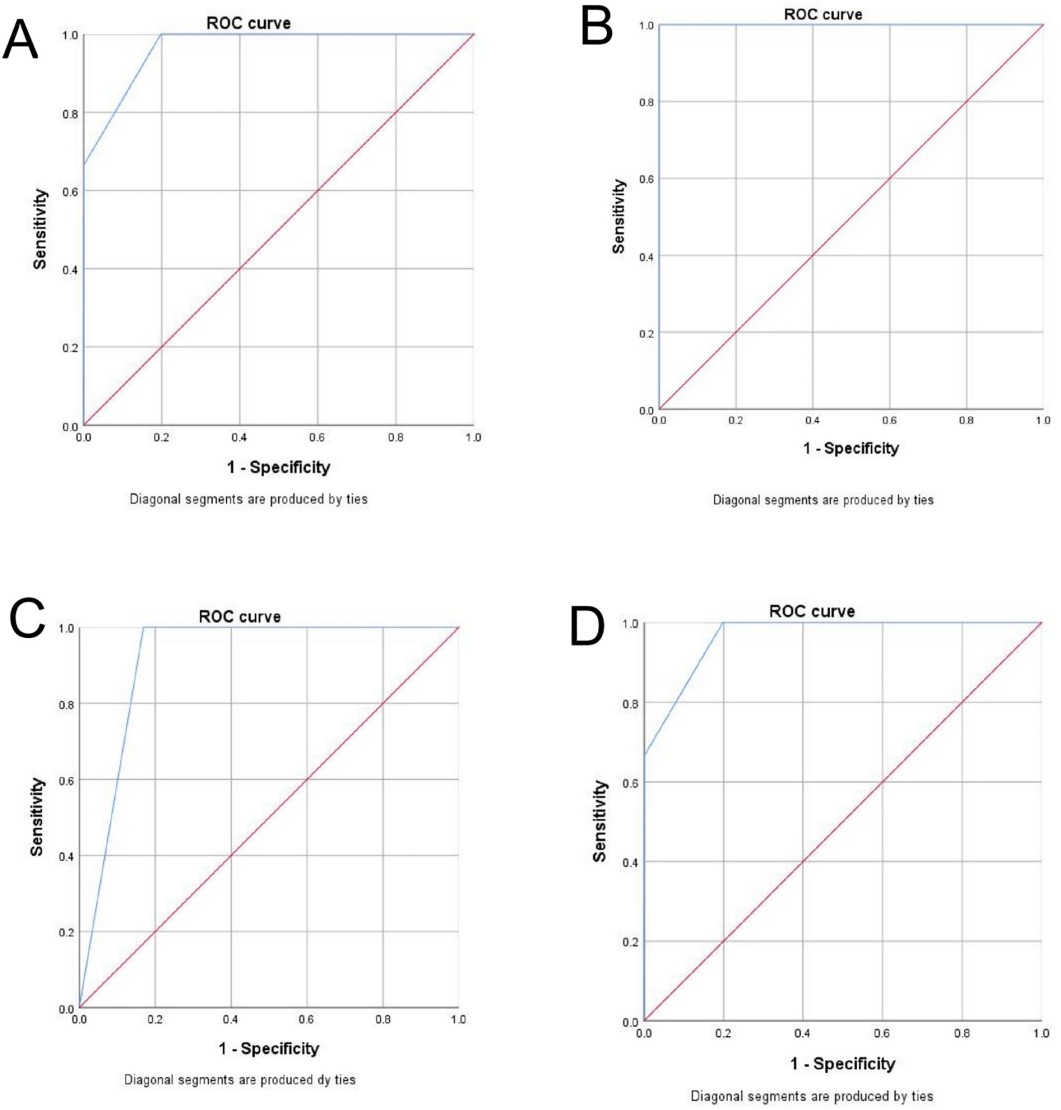
**(A)** The ROC curve of the PAT in predicting hospitalization in pediatric patients. **(B)** The ROC curve of the PAT in predicting critically ill cases in neonatal patients. **(C)** The ROC curve of the PAT in predicting critically ill cases in patients aged 3–8 years old. **(D)** The ROC curve of the PAT in predicting critically ill cases in patients aged 8–15 years old.

**Figure 5:**
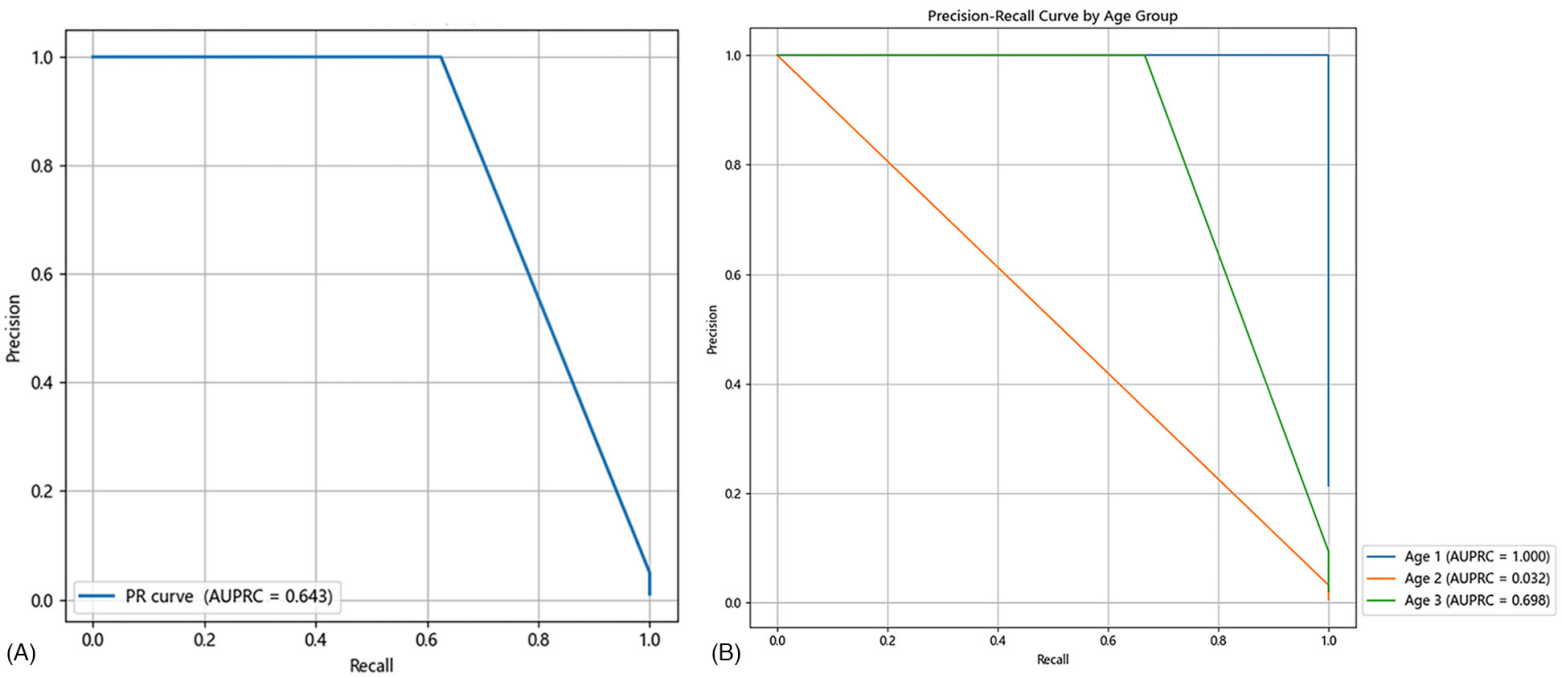
**(A)** The PRC curve of the PAT by logistic function in predicting hospitalization in pediatric patients. **(B)** The PRC curve of PAT by logistic function in predicting hospitalization in pediatric patients by age group (Age 1, neonatal group; Age 2, 3–8 years group; Age 3, 8–15 years group).

The AUROC for PAT in predicting hospitalization in pediatric patients was 0.966, with a sensitivity of 66.7% and a specificity of 99.9% (Figure 4A). The AUPRC by logistic function for PAT in predicting hospitalization in pediatric patients was 0.643 (Figure 5A).

#### 3.4.2 Correlation between the Pediatric Assessment Triangle and critical illness

Among neonatal patients, the AUROC for PAT in predicting critically ill cases was 1.000, with both sensitivity and specificity reaching 100.0% (Figure 4B). In patients aged 3–8 years, the AUROC was 0.944, with a sensitivity of 0.0% and a specificity of 100.0% (Figure 4C). For patients aged 8–15 years, the AUROC was 0.966, with a sensitivity of 50% and a specificity of 99.3% (Figure 4D). The AUPRC by logistic function in neonatal patients was 1.000, while in those aged 3–8 years and 8–15 years, it was 0.032 and 0.698, respectively (Figure 5B).

#### 3.4.3 Waiting time

The mean waiting time for level-2 patients was 3.80 min. All patients received timely and effective treatment or hospital admission, and their condition improved. The mean waiting time for level-3 patients was 14.98 min, and the mean waiting time for level-4 patients was 46.17 min.

#### 3.4.4 Emergency department expenses

The mean cost for level-2 patients was 483.56 yuan, for level-3 patients it was 369.96 yuan, and for level-4 patients it was 352.57 yuan.

## 4 Discussion

With the increasing demand for pediatric visits leading to overcrowding in PEDs, the rational application of emergency pre-triage systems can quickly and efficiently identify critically ill children and ensure the reasonable allocation of medical resources. Compared to adult emergency departments, PEDs have unique characteristics. The young patients are often unable to describe their feelings or assist with their assessment, as they are not simply “miniature versions of adults” (11). However, emphasizing similarities can simplify assessment methods and facilitate disease classification (12). Internationally recognized pediatric triage scales, such as the Pediatric Early Warning Score (PEWS), ATS, ESI, MTS, and paedCTAS, exhibit high reliability and a considerable degree of consistency (7)^.^ The PEWS is one of the most commonly used scoring tools for pediatric pre-triage internationally (13). Research has shown that using the PEWS lacks independent testing validation in PEDs. For example, using the optimal cutoff value to predict PED admissions results in a two- to four-fold increase in pediatric intensive care unit (PICU) admission rates (14).

The PAT is a rapid assessment tool introduced by the American Academy of Pediatrics in 2000, and primarily targets pre-hospital medical services. The PAT is not a diagnostic tool, as its purpose is rather to enable providers to formally express their overall impression of the children, determine the severity of the presentation and the category of pathophysiology, and identify the type and urgency of intervention. As pre-hospital pediatric education continues to evolve, the PAT has become a widely used pediatric assessment tool and an educational training tool for advanced pediatric life-support courses (15). A survey conducted by Benito et al. (16) of emergency healthcare providers who received PAT training showed that 84.9% of emergency physicians continued to use the PAT for the initial assessment of children after receiving PAT training. Moreover, 81.6% of emergency physicians believed that the use of the PAT during the assessment helped establish a diagnosis and that it possessed important clinical significance.

This study reveals the predominance of respiratory diseases in the PED in our center. Of the various types of diseases, the PAT accurately identified the top three critical illnesses as endocrine system diseases (100%), toxic exposure (50%), and circulatory system diseases (40%). Although respiratory emergencies ranked first, the proportion of critical cases among the endocrine, drug poisoning, and circulatory system cases was relatively high, possibly due to the smaller overall totals of such disease types, resulting in a higher proportion of children with relatively severe conditions whose parents sought emergency care for them. For the respiratory system disease cases, the PAT demonstrated 0.0% sensitivity and 100% specificity. The reasons for these results include the limited data in the study, an insufficient number of critical cases, and the rapid changes inherent in respiratory system disease cases, which may have led to changes in their condition during waiting or treatment.

The AUROC for the PAT assessment of hospitalized children was 0.966, and the AUPRC was 0.643, with a sensitivity of 66.7% and a specificity of 99.9%. The relatively low sensitivity may be related to the acceptable condition of the children during pre-triage, changes in their condition during waiting or observation, and fluid infusion. The proportion of critical cases was highest among the neonatal cases (21.4%), which may have been related to the lower tolerance and faster changes in condition associated with neonates. Among the neonates, the AUROC and AUPRC for the PAT for critical cases were 1.000, with a sensitivity and specificity of 100.0%. In the children aged 3–8 years, the AUROC was 0.944, with a sensitivity of 0.0% and a specificity of 100.0%, and in the children aged 8–15 years, the AUROC was 0.966, with a sensitivity of 50% and a specificity of 99.3%. These results reflected the superiority of the PAT in predicting critical cases in neonates. Similar results were also reported by Ma et al. (17), who found that an abnormal PAT was significantly correlated with critical diseases in PEDs, and their AUROC for screening critical children using the PAT was 0.963. Concerning different categories of diseases, the PAT was better at evaluating critical respiratory diseases compared to critical non-respiratory illnesses. For children of different ages, evaluating critical illnesses in children aged 1–36 months using the PAT was superior to that in children aged 3–14 years. The results of this study are thus consistent with the results of Ma et al.'s study.

Fernández et al. (18) conducted a prospective study showing that an initial assessment of high-risk children and their pathophysiologic types, such as respiratory distress, respiratory failure, shock, central nervous system, metabolic diseases, and cardiopulmonary failure, could be conveniently and reliably conducted by pre-examination nurses using the PAT structure. When the PAT was used by trained emergency nurses, abnormal findings were related to hospitalization or admission to the PICU. Gausche-Hill et al. (19) reported that as a rapid assessment tool, the PAT provided convenient and reliable pre-hospital assessments for emergency physicians.

There are presently few extant international studies on waiting time, with a majority of the related studies conducted domestically. This may be due to the large overall population and high numbers of patients seeking medical treatment in China, which has led to a high level of public attention on waiting times. How to shorten waiting times and provide timely medical services to critical patients is thus a current topic of great concern. Hing et al. (20) demonstrated that as their number of patients increased from 2002 to 2009, the average waiting time in US emergency departments increased by 25%, i.e., from 46.5 to 58.1 min; the mean waiting time in our study was significantly less at 39.75 min. The average waiting time for 2-, 3-, and 4-level patients was 3.80, 14.98, and 46.17 min, respectively, which were all within the required waiting time range. In addition, among level-3 patients, individual waiting times could be as long as 72 min, and among level-4 patients, the longest waiting time was 139 min, exceeding the required range. We speculate that this phenomenon was due to a surge in patients during certain periods that far exceeded the reception capabilities of doctors.

This study was based on a proposed 5-level triage system using the PAT and encompassed several aspects of common pediatric emergencies. Level 1 included critically ill children requiring immediate resuscitation in the emergency room. Level 2 included critically ill children with symptoms of stable circulation and cold and wet skin, accompanied by weak peripheral pulsation and unstable vital signs that required monitoring of vital signs in the emergency room and additional treatment. Level 3 was designated for emergency pediatric patients who manifested slight changes in mental status or seizures within 24 h, mild respiratory distress, and ear temperatures ≥39.5°C, requiring priority waiting. Level 4 was for subacute patients who showed stable vital signs and ear temperatures ≥38.5°C, who then received treatment based on arrival time. Level 5 was for non-emergency pediatric patients who were recommended for outpatient follow-up. Gregorio Marañón Hospital proposed a 5-level pediatric triage system based on the PAT called the triage pediatric-Gregorio Marañón system, which was sorted and systematized into six steps, with each step generating a partial priority. The first step was the PAT, which generated a partial priority based on the number of modified sides of the triangle and was applied in the community pediatric emergency services in Madrid (21).

Emergency-department overcrowding is a profound and widespread problem that negatively affects the experience of patients, families, and healthcare providers and requires coordinated efforts across the healthcare system (2). Convenient and efficient emergency pre-triage can reduce emergency department overcrowding to some extent, allowing timely and sequential diagnosis and treatment of pediatric patients. The PAT emergency pre-triage method involved in this study managed the waiting times of the majority of the pediatric patients to within the ideal range, but there remained individuals with prolonged waiting times; therefore, additional effective solutions need to be explored. The concepts of a physician-nurse supplementary triage assessment team (MDRNSTAT) (22) and a triage liaison physician (TLP) (23) have thus been created to expedite patient care based on the medical screening examination needs of emergency-department providers. As verified by a large clinical randomized controlled trial, having a TLP showed superior effectiveness in reducing the waiting time for patients with abdominal pain (24). There have also been reports on the efficacy of implementing the artificial-intelligence, large-language model ChatGPT for rapid diagnosis in emergency departments. The results showed a similarity between diagnoses using ChatGPT and physician diagnoses: the physicians made the correct diagnosis in the top-five differential diagnoses in 83% of cases, while the proportion was 77% for ChatGPT v3.5 and 87% for v4.0. Based on laboratory results, the accuracy of ChatGPT v3.5 (60%) and v4.0 (53%) was also comparable to that of the physicians in selecting the correct primary diagnosis (53%). This study, therefore, revealed that ChatGPT performed similarly to medical expert retrospective evaluations in generating differential diagnoses (25). Accordingly, it can be inferred that the use of large-language models for intelligent pediatric emergency pre-assessment may constitute a new modality for the application of artificial intelligence in clinical scenarios in PEDs.

The present study had some limitations. First, this was a retrospective study without a control group, and detailed data such as the time required for pre-triage, types of pre-triage diseases, and specific abnormal items in the PAT scale were not recorded. Therefore, subsequent prospective controlled studies are needed to standardize the research process, clarify specific indicators, and ensure more comprehensive and accurate research results. Second, this study included relatively meagre data, and there was a certain degree of coincidence; thus, additional expansion of the sample size and random sampling are needed to further ensure the accuracy of the results.

## 5 Conclusions

As a tool to be applied for pre-triage in PEDs, PAT demonstrated significant superiority in the specific identification of critical cases, especially in identifying pediatric respiratory emergencies and neonatal emergencies. Furthermore, the PAT could lead to prompt medical treatment to children at all levels, ensuring the rational allocation of medical resources. We recommend that multicenter studies be conducted in the future to further explore the efficacy of the PAT for triaging in PEDs.

## Data Availability

The original contributions presented in the study are included in the article/Supplementary Material, further inquiries can be directed to the corresponding authors.
